# Oncogenic potential of histone-variant H2A.Z.1 and its regulatory role in cell cycle and epithelial-mesenchymal transition in liver cancer

**DOI:** 10.18632/oncotarget.7194

**Published:** 2016-02-04

**Authors:** Hee Doo Yang, Pum-Joon Kim, Jung Woo Eun, Qingyu Shen, Hyung Seok Kim, Woo Chan Shin, Young Min Ahn, Won Sang Park, Jung Young Lee, Suk Woo Nam

**Affiliations:** ^1^ Laboratory of Oncogenomics, Department of Pathology, College of Medicine, The Catholic University of Korea, Seoul 06591, Republic of Korea; ^2^ Department of Cardiology, College of Medicine, The Catholic University of Korea, Seoul 06591, Republic of Korea; ^3^ Functional RNomics Research Center, College of Medicine, The Catholic University of Korea, Seoul 06591, Republic of Korea; ^4^ Department of Kidney System, College of Oriental Medicine, Kyung Hee University, Seoul 02447, Republic of Korea; ^5^ Cancer Evolution Research Center, The Catholic University of Korea, Seoul 06591, Republic of Korea

**Keywords:** H2A.Z.1, cell cycle, epithelial-mesenchymal transition, liver cancer

## Abstract

H2A.Z is a highly conserved H2A variant, and two distinct H2A.Z isoforms, H2A.Z.1 and H2A.Z.2, have been identified as products of two non-allelic genes, *H2AFZ* and *H2AFV*. H2A.Z has been reported to be overexpressed in breast, prostate and bladder cancers, but most studies did not clearly distinguish between isoforms. One recent study reported a unique role for the H2A.Z isoform H2A.Z.2 as a driver of malignant melanoma. Here we first report that H2A.Z.1 plays a pivotal role in the liver tumorigenesis by selectively regulating key molecules in cell cycle and epithelial-mesenchymal transition (EMT). *H2AFZ* expression was significantly overexpressed in a large cohort of hepatocellular carcinoma (HCC) patients, and high expression of *H2AFZ* was significantly associated with their poor prognosis. H2A.Z.1 overexpression was demonstrated in a subset of human HCC and cell lines. H2A.Z.1 knockdown suppressed HCC cell growth by transcriptional deregulation of cell cycle proteins and caused apoptotic cell death of HCC cells. We also observed that H2A.Z.1 knockdown reduced the metastatic potential of HCC cells by selectively modulating epithelial-mesenchymal transition regulatory proteins such as E-cadherin and fibronectin. In addition, H2A.Z.1 knockdown reduced the *in vivo* tumor growth rate in a mouse xenograft model. In conclusion, our findings suggest the oncogenic potential of H2A.Z.1 in liver tumorigenesis and that it plays established role in accelerating cell cycle transition and EMT during hepatocarcinogenesis. This makes H2A.Z.1 a promising target in liver cancer therapy.

## INTRODUCTION

Genomic DNA in eukaryotic cells is packaged into chromatin, with the nucleosome being the smallest subunit that contains canonical histones: H2A, H2B, H3, H4, and H1. These histones are assembled in H3-H4 heterodimer and H2A-H2B heterodimer with the linker histone H1 holding the nucleosome together [1]. The highly dynamic changes in nucleosome composition and in their biochemical properties allow for regulation of transcription, gene silencing, DNA replication and recombination [2]. H2A.Z is one of the histone H2A variants that has 60% similarity with canonical histone H2A in mammal cells [3] and that functions relative to chromatin remodeling, gene transcription regulation, chromosome segregation and DNA repair [4, 5]. H2A.Z exchange is promoted by ATP-dependent exchange complexes, including Snf2-Related CREBBP activator (SRCAP) [6] and p400/Tip60 complex [7], and it plays a major role in critical biological processes, such as chromosome segregation [8], cell cycle progression [9] and maintaining heterochromatin/euchromatin status [10]. It is so far the only histone that has been shown to be indispensable for survival in many organisms, and although only a single gene copy is present in invertebrates, two distinct gene copies exist in vertebrates [11]. These encode two functionally different H2A.Z.1, encoded by *H2AFZ*, and H2A.Z.2 subtypes, encoded by *H2AFV* [12] that have recently been implicated in various cancers [13]. While they differ by only three amino acids at the protein level, H2A.Z.1 and H2A.Z.2 are encoded by distinct nucleotide sequences. Currently, isoform-specific functions remain largely unclear, and H2A.Z.1 mouse knockout studies suggest that the two genes are non-redundant, which could suggest a structural difference between them; additionally, preliminary data indicate that they may impart nucleosomes with different structural and functional properties [11]. For example, a preferential increase of H2A.Z.1 was observed in castration-resistant lymph node carcinoma of the prostate xenograft tumors (a form of androgen-independent tumor) [14]. However, more direct experimental evidence in support of the structural and functional differences imparted by these two H2A.Z variants is still required. Moreover, in the context of tumorigenesis, H2A.Z is overexpressed in breast, prostate, and bladder cancers, where in some cases, it increases proliferation [13]. However, these studies either focused solely on H2A.Z.1, or did not clearly distinguish between isoforms.

A recent study reported a unique role for the H2A.Z isoform H2A.Z.2 as a driver of malignant melanoma [15]. H2A.Z.2 is highly expressed in metastatic melanoma, correlates with decreased patient survival and is required for cellular proliferation that implicates H2A.Z.2 as a mediator of cell proliferation and drug sensitivity in malignant melanoma; these findings hold translational potential for novel therapeutic strategies. However, in the present study, in contrast to metastatic melanoma, we report a distinct role for H2A.Z.1 in human liver cancer. H2A.Z.1 is significantly overexpressed in a large cohort of HCC patients and correlates with their poor prognosis. H2A.Z.1 also promotes proliferation by selectively regulating cell cycle components. We further identified EMT protein, E-cadherin and fibronectin as H2A.Z.1 regulatory proteins whose levels are also elevated in a large cohort of HCC patients. Hence, our studies suggest that oncogenic potential of H2A.Z.1 in hepatocarcinogenesis by selectively modulating cell cycle and EMT regulatory proteins and that targeting H2A.Z decomposition may be an emerging therapy for the molecular treatment of liver malignancy.

## RESULT

### H2A.Z.1 is aberrantly overexpressed in HCC patients, and its expression is associated with their poor prognosis

**Figure 1:**
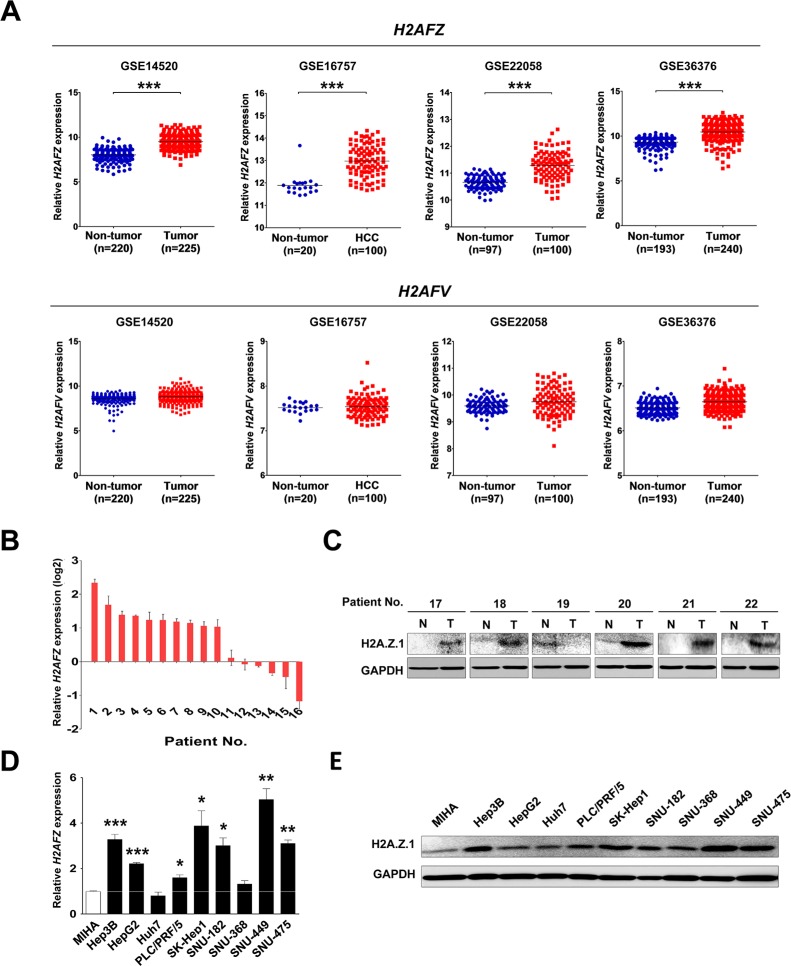
Aberrant expression of H2A.Z.1 in a human HCC (**A**) An analysis of microarray data from the Gene Expression Omnibus (GEO) database. GEO data sets of GSE14520, GSE16757, GSE22058 and GSE36376 showed that *H2AFZ* expression was significantly overexpressed in HCC compared with a non-tumor group. Upper panel: *H2AFZ* mRNA expression, lower panel: *H2AFV* mRNA expression (mean ± S.D., ***P < 0.001, versus Non-tumor). (**B**) The mRNA expression of *H2AFZ* was performed by qRT-PCR in a subset of HCC tissues. (**C**) A western blot analysis of H2A.Z.1 for a subset of HCC tissues (T: tumor tissues; N: adjacent noncancerous tissues). Glyceraldehyde-3-phosphate dehydrogenase (GAPDH) is shown as a loading control. (**D**) The qRT-PCR analysis of *H2AFZ* in ten hepatic cell lines, including one normal (MIHA) and nine hepatoma cell lines (Hep3B, HepG2, Huh7, PLC/PRF/5, SK-Hep1, SNU-182, SNU-368, SNU-449, and SNU-475). GAPDH was for normalization (mean ± S.D., *n* = 3, *P < 0.05, **P < 0.01, versus MIHA cell). (**E**) The endogenous protein expressions of H2A.Z.1 in hepatic cell lines were analyzed through a western blot analysis. GAPDH is indicated as a loading control.

Very recently, we noted a report that established a unique role for H2A.Z.2 in driving melanoma cell proliferation and drug sensitivity [15]. For liver cancer, neither aberrant expression of H2A.Z nor correlation with clinicopathlogical features of HCC was reported. Thus, the functional role of H2A.Z in liver cancer has yet to be uncovered, and it would be of interest to learn if H2A.Z.2 plays a similar role in liver cancer. Therefore, to evaluate the expression of H2A.Z in liver cancer, we recapitulated both *H2AFZ* and *H2AFV* expressions in the large cohorts of HCC patients that were available from the National Center for Biotechnology Information (NCBI) and Gene Expression Omnibus (GEO) database (accession numbers GSE14520, GSE16757, GSE22058 and GSE36376), and the data are presented as scatter plots. Unlike with previous observations of metastatic melanoma, only *H2AFZ* was significantly overexpressed in these four different HCC cohorts, whereas *H2AFV* expression was not changed in HCC (Figure 1A, Supplementary Table S1). Next, to verify the overexpression of *H2AFZ* in HCC patients, *H2AFZ* expressions of 16 randomly selected HCC tissues paired with adjacent non-cancerous liver tissues were investigated by quantitative real-time PCR (qRT-PCR). From this, 10 out of 16 HCC tissues exhibited significant overexpression of *H2AFZ* in HCC (Figure 1B). Consistently, increased expression of H2A.Z.1 protein was confirmed by immunoblotting of 6 randomly selected human HCC tissues with corresponding non-cancerous liver tissues (Figure 1C). Additionally, endogenous expression of *H2AFZ* was investigated by qRT-PCR in 10 different liver cell lines, including immortalized normal hepatic cell line, MIHA (Figure 1D). The human liver cancer cell lines (Hep3B, HepG2, PLC/PRF/5, SK-Hep-1, SNU-182, SNU-449 and SNU-475) exhibited relatively high *H2AFZ* expression levels compared with that of immortalized normal hepatic cell line, MIHA. Human liver cancer cell lines also exhibited relatively high expression of H2A.Z.1 protein compared with MIHA cells (Figure 1E). To generalize our finding in an animal model, we recapitulated both *H2AFZ* and *H2AFV* expressions from the three different mouse liver cancer study data sets that were available from the NCBI GEO database (accession numbers GSE29813, GSE35289, and GSE57597). In agreement with human results, the mouse liver cancer GEO data sets showed that only *H2afz* (a mouse form of *H2AFZ* gene) was significantly overexpressed in mouse liver cancer (Supplementary Figure S1).

**Figure 2:**
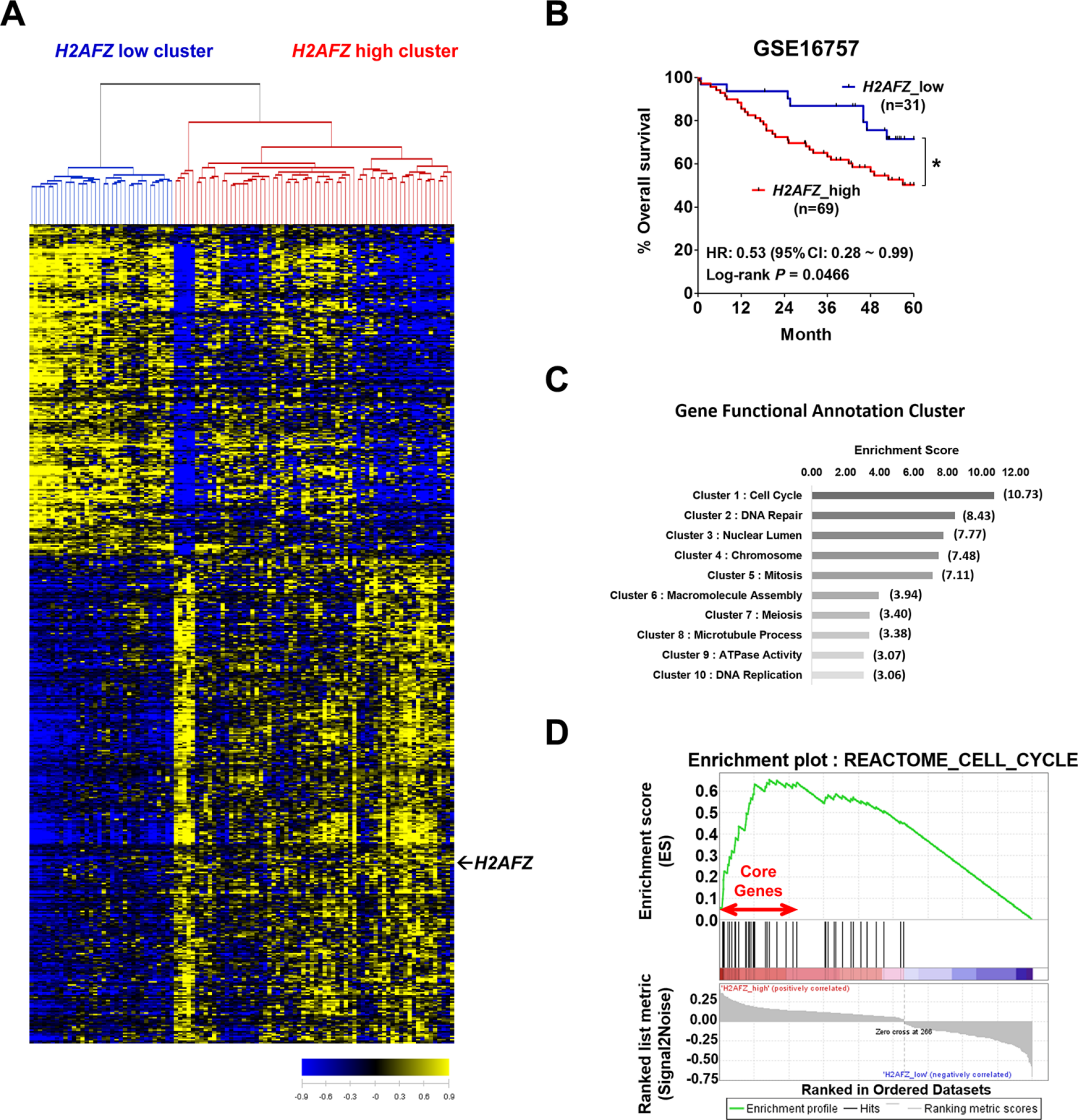
Clinical relevance of *H2AFZ* expression in HCC patients and cluster analysis of *H2AFZ*-associated genes (**A**) The microarray data were obtained from the GEO database (GSE16757). 524 genes were selected for cluster analysis (*P* < 0.01, *r* > 0.4 or *r* < −0.4). Patients were divided into *H2AFZ* low cluster and *H2AFZ* high cluster. (**B**) Kaplan-Meier survival curves for overall survival. *P*-values were obtained with the log-rank test. (**C**) Correlated genes with *H2AFZ* were subjected to DAVID, a molecular function mining tool. The bar graph shows a gene enrichment score of correlation gene annotation clusters with *H2AFZ*. (**D**) GSEA analysis of *H2AFZ*-specific molecular signature. A growth-related gene set (REACTOME_CELL_CYCLE) enriched to *H2AFZ* signatures is shown; the barcode indicates gene positions. The y-axis indicates the extent of enrichment.

Because mRNA and protein levels of H2A.Z.1 showed up-regulation in HCCs, we next assessed the prognostic association of H2A.Z.1 expression in a large cohort of 100 HCC patients (GSE16757). A significant number, 524, of genes with expression patterns that highly correlated with H2A.Z.1 expression were selected for cluster analysis (*P* < 0.001, *r* > 0.4 or *r* < −0.4) and are shown as heatmaps (Figure 2A). Patients were then divided into the following two groups: H2A.Z.1 high cluster (*H2AFZ*_high) and H2A.Z.1 low cluster (*H2AFZ*_low). The Kaplan-Meier survival curves of patients with HCC indicated that the 5-year overall survival rate of patients with high H2A.Z.1 expression was significantly lower than that of patients with low H2A.Z.1 expression (*P* = 0.0466; Figure 2B). Molecular hepatocarcinogenesis has been reported to be different according to etiologies such as hepatitis B virus, hepatitis C virus, alcohol, etc. However, additional GEO data (GSE50579 and GSE16757) analysis showed no difference of H2A.Z.1 expression according to such etiologies (Supplementary Figure S2). These results suggest that the expression of H2A.Z.1 is up-regulated during hepatocarcinogenesis and that its high expression is associated with the biological process of tumorigenesis and poor prognosis of HCC patients.

**Figure 3:**
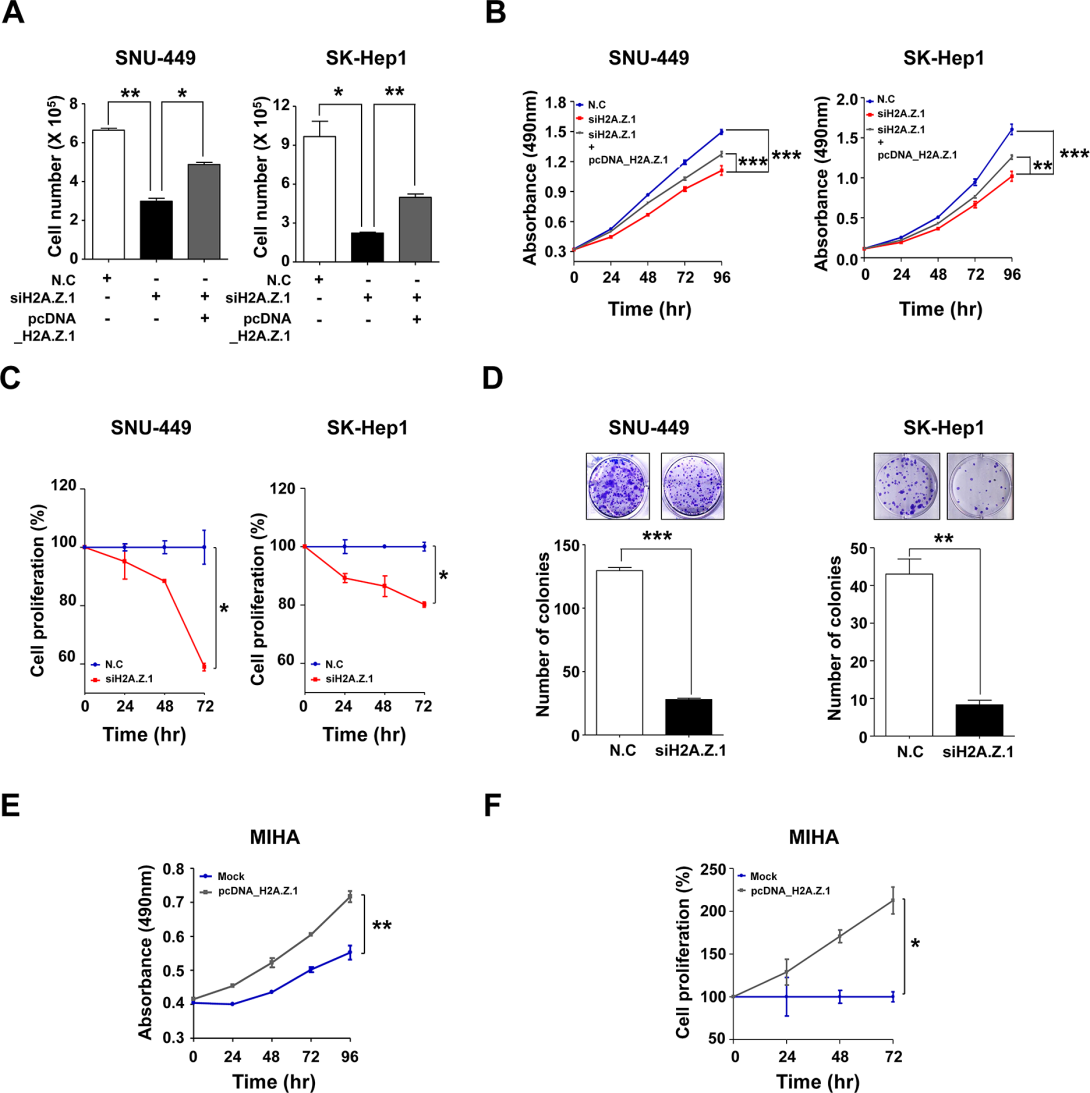
Effect of H2A.Z.1 knockdown on cell growth and cell proliferation (**A**) SNU-449 and SK-Hep1 cell lines were transfected with negative control siRNA (N.C, 100 nM) or H2A.Z.1-specific siRNA (siH2A.Z.1, 100 nM) or H2A.Z.1 expressing plasmid (pcDNA3.1_H2A.Z.1). After 72 hr incubation, the cell number was determined using trypan blue cell counting (mean ± S.D., *n* = 3, *P < 0.05, **P < 0.01). (**B**) SNU-449 and SK-Hep1 cell lines were transfected with negative control siRNA (N.C, 100 nM) or H2A.Z.1-specific siRNA (siH2A.Z.1, 100 nM) or H2A.Z.1 expressing plasmid (pcDNA3.1_H2A.Z.1). Cell growth was measured by MTS assay over a period of 96 hrs. All experiments were performed in triplicate (mean ± S.D., *n* = 3, **P < 0.01, ***P < 0.001). (**C**) Bromodeoxyuridine (BrdU) incorporation assays were carried out using SNU-449 and SK-Hep1 cell lines (mean ± S.D., *n* = 3, *P < 0.05). (**D**) Clonogenic assays were performed in SNU-449 and SK-Hep1 cell lines. Upper panel: representative colony images. Lower panel: three randomly selected images shown on graphs (mean ± S.D., *n* = 3, **P < 0.01, ***P < 0.001). (**E**) MIHA cell lines was transfected with Mock plasmid (pcDNA3.1_Mock) or H2A.Z.1 expressing plasmid (pcDNA3.1_H2A.Z.1). Then cell growth rate was measured by MTS assay over a period of 96 hrs. All experiments were performed in triplicate (mean ± S.D., *n* = 3, **P < 0.01). (**F**) Bromodeoxyuridine (BrdU) incorporation assays were carried out using MIHA cell lines (mean ± S.D., *n* = 3, *P < 0.05).

Next, to investigate the biological relevance of the *H2AFZ*-associated molecular signature, we subjected these gene clusters (524 genes) to the DAVID Web-based bioinformatics platform (http://david.abcc.ncifcrf.gov/) to retrieve the biological pathways of the *H2AFZ* signature. The resultant “Gene Functional Annotation Cluster” mapping as a part of the DAVID tool showed the biological process to map molecular data sets (Figure 3C). The major signaling pathways that recapitulated in *H2AFZ* signature were Cell cycle, DNA repair, Nuclear lumen, Chromosome, Mitosis, Macromolecule assembly, Meiosis, Microtubule process, ATPase activity and DNA replication (Figure 2C, Supplementary Table S2). To better understand the molecular mechanism of H2A.Z.1 in hepatocarcinogenesis, we next performed gene set enrichment analysis (GSEA) using an *H2AFZ* signature to identify *H2AFZ*-related gene sets. GSEA identified intracellular signaling pathways enriched in the *H2AFZ* signature of HCC, and REACTOME_CELL_CYCLE was identified as a highly significant enriched gene set in *H2AFZ* signature (Figure 2D, Supplementary Table S3).

### Targeted inhibition of H2A.Z.1 elicits a tumor-suppressor effect by regulating cell cycle and EMT proteins in liver cancer cells

**Figure 4:**
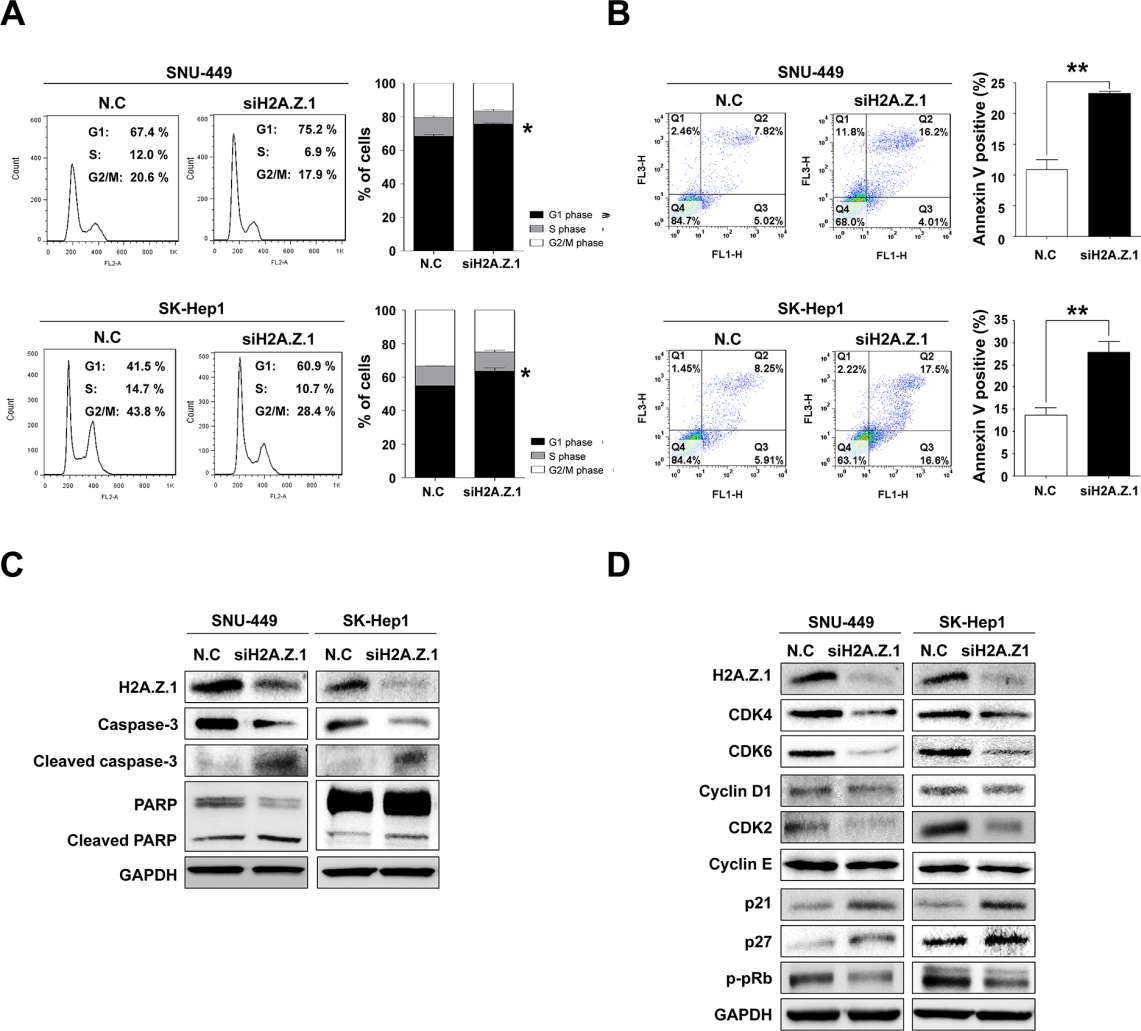
Targeted disruption of H2A.Z.1 caused cell death and cell cycle arrest in liver cancer cells (**A**) Cell cycle analysis. FACS analysis was conducted after negative control siRNA or H2A.Z.1 siRNA transfection. When H2A.Z.1 knockdown was induced, G1/S arrest occurred in SNU-449 and SK-Hep1 cell lines. The percentage indicates the distribution of the cells (mean ± S.D., *n* = 3, *P < 0.05). (**B**) Apoptosis analysis. Negative control siRNA (N.C) or H2A.Z.1 siRNA (siH2A.Z.1) were treated in SNU-449 and SK-Hep1 cell lines. After 72 hr, fluorescence-activated cell sorting analysis (FACS) was performed using PI (FL3-H) and annexin V (FL1-H) staining. The bar graphs indicated the percentage of annexin V positive cells (mean ± S.D., *n* = 3, **P < 0.001). (**C**) Western blot analysis of caspase-3, cleaved caspase-3, PARP, and cleaved PARP showed that H2A.Z.1 knockdown induced apoptosis in SNU-449 and SK-Hep1 cell lines. GAPDH was used as a loading control. (**D**) Western blot analysis. Negative control siRNA (N.C) or H2A.Z.1 siRNA (siH2A.Z.1) were treated in SNU-449 and SK-Hep1 cell lines. The protein levels of H2A.Z.1, CDK4, CDK6, Cyclin D1, Cyclin E, p21, p27, CDK2 and phosphorylated-pRb (p-pRb) were detected with their specific antibodies. GAPDH was used as the endogenous loading control.

To better understand the molecular functions of H2A.Z.1 in liver tumorigenesis, H2A.Z.1 knockdown was attempted by way of RNA-interference and studied in cell viability, MTS cell proliferation, BrdU incorporation and clonogenic assays. H2A.Z.1 knockdown resulted in reduced growth and proliferation rates of the SNU-449 and SK-Hep1 liver cancer cells (Figure 3A–3D). In contrast, the suppressive effect of H2A.Z.1 knockdown on cell growth was significantly rescued by the co-transfection of H2A.Z.1 expressing plasmid (pcDNA_H2A.Z.1) in the same cells (Supplementary Figure S3A and Figure 3A and 3B). In accordance with these results, we also observed that ectopic expression of H2A.Z.1 protein augmented cellular growth and proliferation of the normal hepatic cell line, MIHA (Supplementary Figure S3B and Figure 3E and 3F). The anti-growth effect could be partially explained by the disruption of cell growth regulation, such as cell cycle arrest, cellular senescence, or apoptosis, on H2A.Z.1-targeting. Flow cytometric cell cycle analysis indicated that H2A.Z.1 knockdown led to a significant increase in the number of cells in the G1 phase with a concomitant decrease in the number of cells in the S phase and G2/M phase in both SNU-449 and SK-Hep1 liver cancer cell lines (Figure 4A). We then stained the cells with annexin V-FITC and PI after transfection of H2A.Z.1-targeting siRNA (siH2A.Z.1) for apoptosis analysis. As expected, H2A.Z.1 knockdown caused a significant induction of apoptotic cells compared with negative control siRNA (N.C) and induced both caspage-3 and PARP cleavages in SNU-449 and SK-Hep1 liver cancer cell lines (Figure 4B and 4C). Further, because GSEA suggested REACTOME_CELL_CYCLE as a highly significant enriched gene set in *H2AFZ* signature, we next performed western blot analysis for cell cycle regulatory proteins to clarify the underlying mechanism of the growth inhibition elicited by H2A.Z.1-targeting. Western blot analysis showed that H2A.Z.1 knockdown suppressed the expression of CDK4, CDK6, cyclin D1 and CDK2, and simultaneously induced p21^WAF1/Cip1^ and p27^Kip1^ in both SNU-449 and SK-Hep-1 cells (Figure 4D). These results strongly suggest that H2A.Z.1 overexpression causes the suppression of the negative cell cycle modulators such as p21^WAF1/Cip1^ and p27^Kip1^ and at the same time, induces the expression of their specific regulators such as CDK4, CDK6, cyclin D1, CDK2 in the cell cycle transition of liver cancer cell. In general, activated cyclin/CDK complex can cause hyperphosphorylation of pRb which loses its tumor suppressor activity and which allows for E2F/DP1 transcriptional activity. Thus, we next investigated whether the dysregulation of cyclins and CDKs by H2A.Z.1-targeting affects the E2F/DP1 transcriptional activity. As expected, targeted disruption of H2A.Z.1 caused hypophosphorylation of pRb in both SNU-449 and SK-Hep-1 cells. This result implies that the aberrant regulation of H2A.Z.1 affects the phosphorylation of Rb protein via transcriptional activation of cyclin/CDKs and/or inactivation of negative modulators in liver tumorigenesis.

**Figure 5:**
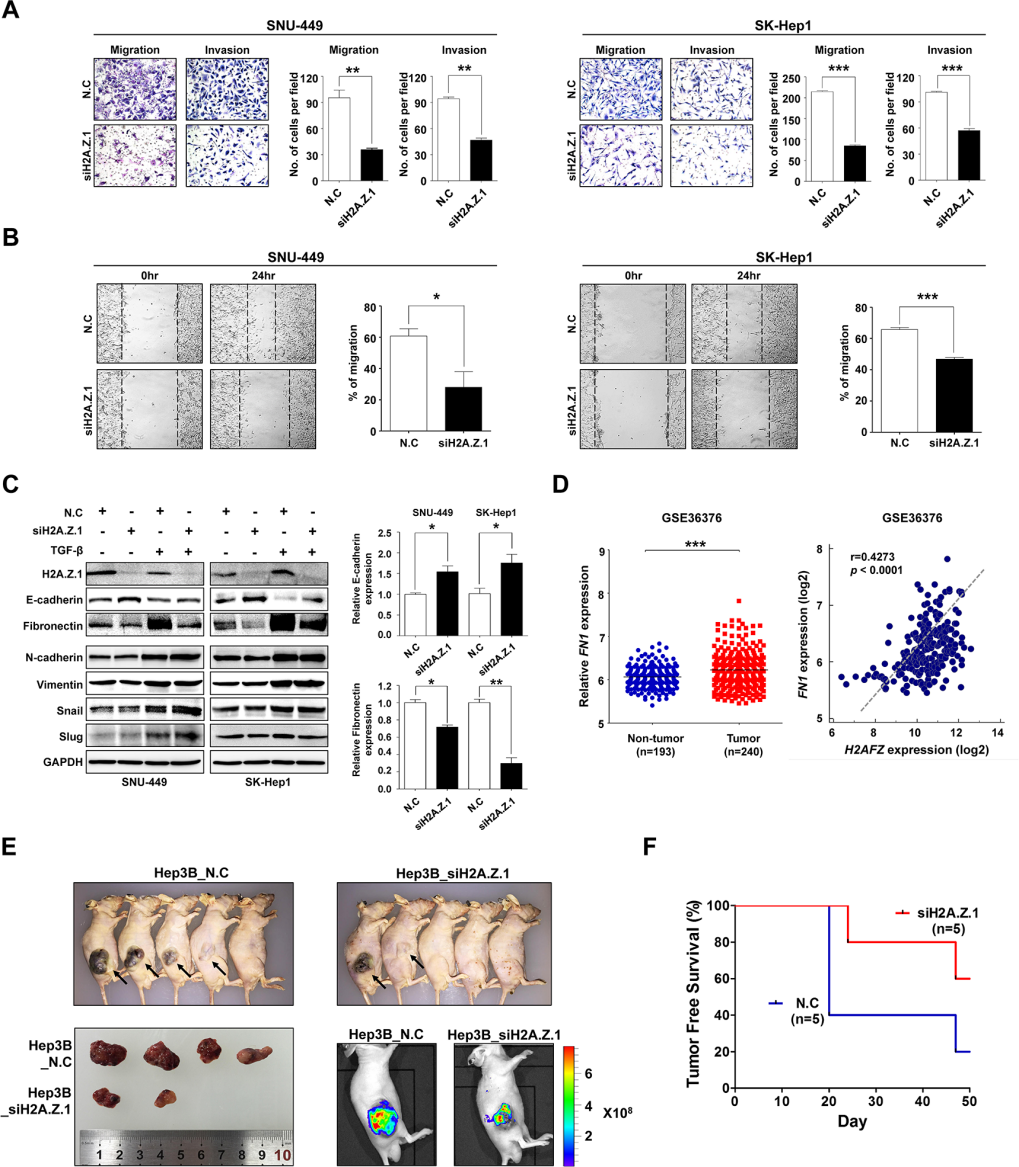
Repression of H2A.Z.1 expression inhibited the metastatic potential of liver cancer cells (**A**) The effect of H2A.Z.1 knockdown on *in vitro* cell migration and invasion. H2A.Z.1 knockdown inhibited migration and invasion of SNU-449 and SK-Hep1 cell lines. The cell number was determined by migrated and invaded cells. Three randomly selected fields were captured and presented by as a graph (mean ± S.D., *n* = 3, **P < 0.01, ***P < 0.001). The Figure images are representative images. (**B**) A scratch wound healing assay. A scratch in the plate on which the cell seeding was performed and incubated for 24 hrs. The bar graphs show the ratio of a recovered area (mean ± S.D., *n* = 3, ***P < 0.001, *P < 0.05). (**C**) A western blot analysis for EMT markers. The protein levels of H2A.Z.1, E-cadherin, fibronectin, N-cadherin, vimentin, snail and slug were detected with their specific antibodies. The GAPDH was made use for a loading control. Densitometry was used to quantify western blot data (mean ± S.D., *n* = 3, ***P < 0.001, *P < 0.05). (**D**) An analysis of microarray data from the Gene Expression Omnibus (GEO) database. A GEO data set of the accession numbers GSE36376 was used for expression analysis between *H2AFZ* and *FN1*. Left panel, *FN1* mRNA expression in GSE36376. Right panel, positive correlation of *H2AFZ* and *FN1* expression in GSE36376 (Pearson correlation coefficient *r* = 0.4273, ***P < 0.0001). (**E**) Tumor growth of animals injected with Hep3B transfected siH2A.Z.1 siRNA (Hep3B_siH2A.Z.1) or negative control siRNA (Hep3B_N.C). Images obtained at sacrifice after 50 days of mice (upper), tumor (lower left) and fluorescent image (lower right). (**F**) Kaplan-Meier tumor-free survival curves. The curve shows tumor-free survival of total 10 mice.

Next, to gain further insights into the biologic roles of H2A.Z.1in hepatocarcinogenesis, we integrated a comprehensive collection of cancer-related gene expression signatures and analyzed the H2A.Z.1 signature using the molecular concept map (ConceptGen) [16]. This analysis allowed us to navigate a network of associations that involved H2A.Z.1-related gene elements and to identify an enrichment network that linked the H2A.Z.1 signature with cell cycle, cell migration, apoptosis and chromosome regulation programs (Supplementary Figure S4). From this, we noted that the signature of H2A.Z.1 was highly linked to cell migration or cell migration–related signatures. Thus, to elucidate the role of H2A.Z.1 in the malignant behavior of liver cancer cells, we performed *in vitro* motility and invasion assays. A modified Boyden chamber assay revealed that H2A.Z.1 knockdown significantly suppressed chemoattractant (5% fetal bovine serum)-stimulated migratory and invasive responses of both SNU-449 and SK-Hep-1 cells (Figure 5A). Similarly, a scratch wound healing assay also showed that H2A.Z.1 knockdown reduced chemoattractant-stimulated wound-healing efficacy of same liver cancer cells (Figure 5B). To clarify the regulatory effect of H2A.Z.1 on EMT, western blot analysis was performed for the EMT regulatory proteins in liver cancer cells. Notably, fibronectin was dramatically decreased in H2A.Z.1 knockdown SNU-449 and SK-Hep-1 cells, whereas E-cadherin was increased in same cells (Figure 5C). EMT regulatory molecules are usually influenced by TGF-β signaling pathway [17]. Thus, to clarify that H2A.Z.1 selectively regulates E-cadherin and fibronectin among EMT molecules in liver cancer cells, we performed western blot analysis for EMT molecules in the presence or absence of TGF-β (5 ng/ml) in SNU-449 and SK-Hep1 cells. We observed that TGF-β treatment induced EMT activation and regulated E-cadherin and fibronectin in SNU-449 and SK-Hep1 cells (Supplementary Figure S5 and Figure 5C). Notably we also observed that H2A.Z.1 knockdown suppressed TGF-β-induced EMT activation via selective regulation of E-cadherin and fibronectin proteins in the same cells. Furthermore, gene expression analysis of a large cohort of HCC patients from the NCBI GEO database (accession number GSE36376) consistently showed that fibronectin gene expression was significantly up-regulated in HCC and also showed a positive correlation with H2A.Z.1 in the same cohort study (Figure 5D). These results suggest that the metastatic potential of H2A.Z.1 could be attributed to the selective regulation of EMT proteins, such as E-cadherin and fibronectin, in liver cancer cells. Lastly, to demonstrate whether H2A.Z.1 knockdown has a tumor suppressive effect *in vivo*, we subcutaneously injected H2A.Z.1 knockdown cells (Hep3B_siH2A.Z.1) into athymic nude mice. From this, we found that overall tumor growth rate and average volume at sacrifice were significantly reduced in H2A.Z.1 knockdown Hep3B cell lines compared to control (Figure 5E). In addition, at 50 days post-inoculation, tumors were detected in 4 out of 5 animals injected with control cells (Hep3B_N.C), whereas tumors were observed in 2 out of 5 animals injected with H2A.Z.1 knockdown cells (Figure 5F).

## DISCUSSION

Histone variants appear to play a major role in gene expression regulation. Specifically, the histone H2A variant H2A.Z has been shown to be deregulated in some tumors, causing aberrant expression of a number of oncogenes [18]. H2A.Z.1 and H2A.Z.2 are distinct isoforms of H2A.Z, which has an established role in regulating transcription and is overexpressed in numerous tumor types. However, most studies did not clearly distinguish between isoforms. In this regard, a recent study reported a unique role for the H2A.Z isoform H2A.Z.2 as a driver of malignant melanoma [15]. H2A.Z.2 is highly expressed in metastatic melanoma, correlates with decreased patient survival, and promotes and maintains BRD2, E2F1, and histone acetylation levels to drive melanoma proliferation, which suggests a potential epigenetic therapeutic strategy to improve drug sensitivity in malignant melanoma. Thus, we aimed to characterize the role of H2A.Z deregulation in liver tumorigenesis and determine which H2A.Z isoform might be involved in this process. In the present study, we report a distinct role for H2A.Z.1 human liver cancer. Unlike H2A variant H2A.Z.2 in metastatic melanoma, we found that H2A.Z.1 is solely overexpressed in a large cohort of HCC patients and correlates with their poor prognosis. Our results suggest oncogenic potential of histone-variant H2A.Z.1 and its regulatory role in cell cycle and EMT in liver cancer.

H2A.Z has been reported to be aberrantly overexpressed in many cancers, including breast, prostate, bladder, and colorectal cancer. H2A.Z is enriched in euchromatin, acting as a proto-oncogene with established roles in hormone responsive cancers and overexpressed in endocrine-resistant disease. For instance, in breast cancer, H2A.Z enrichment of the Myc gene promoter region induces elevated Myc expression. Positive feedback between H2A.Z and Myc facilitates breast cancer tumorigenesis [19]. Meanwhile, it has been reported that sirtuin 1 and H2A.Z deregulation in prostate cancer are reciprocally related, and that impaired sirtuin 1-mediated downregulation of H2A.Z via proteasome-mediated degradation. [20]. However, these studies either focused solely on H2A.Z.1 or did not clearly distinguish between isoforms. Therefore, isoform-specific functions remain to be elucidated. In this respect, we noted a recent report that suggested a melanoma-specific role for H2A.Z.2 in promoting proliferation, and we also assumed that it would be of interest to learn if H2A.Z.2 plays a role in other tumors, such as liver cancer. A melanoma study showed that H2A.Z.1 and H2A.Z.2 were expressed at higher levels in melanoma compared with benign nevi and that higher expression correlated with lower survival in patients with metastatic melanoma [15]. However, our result indicated that H2A.Z.1 was significantly overexpressed in HCC compared with corresponding non-cancerous surrounding liver tissues, whereas H2A.Z.2 expression exhibited no changes between tumor vs non-tumor, and higher expression of H2A.Z.1 correlated with lower survival in patients with HCC (Figures 1 and 2). Further, in a melanoma study, knockdown of H2A.Z.2 induced G1/S cell cycle arrest in melanoma cells. Transcriptional profiling revealed that H2A.Z.2 positively regulates the expression of E2F target genes involved in cell-cycle progression. In contrast, our results demonstrated that H2A.Z.1 knockdown induced G1/S cell cycle arrest and apoptotic cell death. H2A.Z.1 depletion selectively suppressed positive G1/S cell cycle components, such as CDK4, CDK6, cyclinD1 and CDK2, and simultaneously induced the expression of potent negative cell cycle regulators, p21^WAF1/Cip1^ and p27^Kip1^, in liver cancer cells (Figure 4). Intriguingly, H2A.Z.1 depletion also repressed the metastatic potential of liver cancer cells via selectively modulating EMT regulatory proteins such as E-cadherin and fibronectin (Figure 5). These results imply that H2A.Z.1 may contribute to hepatocellular malignant transformation and proliferation by way of transcriptional activation of cell cycle or EMT components during liver tumorigenesis.

Taken together, in the present study, we showed that H2A.Z.1 is up-regulated in HCCs and that targeted inactivation of H2A.Z.1 inhibits *in vitro* liver tumorigenesis by selectively modulating cell cycle and EMT regulatory proteins in liver cancer cells, which suggested that the development of specific H2A.Z.1 modulators may be crucial for helping to control tumor progression or even for reversing cancer phenotype. These findings define a central role for H2A.Z.1 in liver tumorigenesis and provide a novel strategy for therapeutic intervention in liver cancer.

## MATERIALS AND METHODS

### Tissue samples

Sixteen and 6 pairs of HCC tissues with their corresponding non-cancerous liver tissues were obtained from the archives of the Department of Pathology, Yonsei University (Seoul, Korea) and from the Liver Cancer Specimen Bank of the National Research Resource Bank Program of the Korea Science and Engineering Foundation of the Ministry of Science and Technology. Written informed consent was obtained from each subject according to the Declaration of Helsinki, and the study was approved by the Institutional Review of Board (IRB) of the College of Medicine (Songeui Campus) of the Catholic University of Korea (IRB approval number: MC12SNMI0184).

### Cell culture and treatment

Hep3B, HepG2, Huh7, PLC/PRF/5, SK-Hep1, SNU-182, SNU-368, SNU-449 and SNU-475 HCC cell lines were acquired from KCLB (Korean Cell Line Bank, Seoul, South Korea). MIHA normal liver cell lines were purchased from ATCC (Manassas, VA, USA). All of the cell lines were held in an RPMI-1640 or DMEM medium (Lonza, Walkersville, MD) with 10% fetal bovine serum added (Sigma, ST. Louis, MO) and 100 units/ml of penicillin-streptomycin (Invitrogen, Carlsbad, CA, USA). All cells were cultured at 37°C in a humidified incubator with 5% CO_2_. The human recombinant TGF-β was purchased from R & D Systems (Minneapolis, MN). The TGF-β was dissolved in an aqueous solvent (vehicle) containing 4 mM HCl and 1 mg/ml BSA. The cells were starved in medium containing 0.1% FBS for 4 hr and then treated with TGF-β (5 ng/ml) or vehicle followed by cell lysate preparation.

### Plasmid construction and transfection

The H2A.Z.1 overexpression plasmid was purchased Genscript (Piscataway, NJ, USA). And the lipofectamine 2000 was used for plasmid transfection, according to the manufacturer's instructions (Invitrogen, Carlsbad, CA, USA).

### Gene expression data

To analyze the expression levels of *H2AFZ, H2AFV* mRNAs in HCC, gene expression profiling data sets were obtained from the National Center for Biotechnology Information (NCBI) Gene Expression Omnibus (GEO) databases (Accession Nos. GSE14520, GSE16757, GSE22058, GSE29813, GSE35289, GSE36376 GSE57597).

### Western blot analysis

Cells were lysed using a lysis buffer (50 mM HEPES, 5 mM EDTA, 50 mM NaCl, 1% Triton X-100, 50 mM NaF, 10 mM Na2P4O7, 1 mM Na3VO4, 5 ug/mL aprotinin, 5 ug/mL leupeptin, 1 mM PMSF and protease inhibitor cocktail). Lysates containing equal amounts of proteins were separated by SDS-PAGE and transferred onto a polyvinylidene difluoride membrane (Bio-Rad, Hercules, CA). The blots were blocked with a 5% skim milk solution and incubated with the following antibodies: anti-H2A.Z.1, anti-p21, anti-PARP, anti-caspase-9, anti-caspase-3, anti-cleaved caspase-3, anti-p27, anti-Cyclin D1, anti-Cyclin A2, anti-CDK2, anti-p-pRb, anti-vimentin, and anti-slug (Cell Signaling Technology, Danvers, MA), anti-GAPDH, and anti-Fibronectin (Santa Cruz Biotechnology, Santa Cruz, CA), anti-E-cadherin, and anti-N-cadherin (BD Transduction, San Jose, CA), and anti-Snail (Abcam, Cambridge, MA). The Immobilon^™^ western blot detection system (Millipore, Billerica, MA) was used to detect bound antibodies. The intensities of the western blot bands were quantified using LAS-3000 (Fuji Photo Film Co., Japan).

### Cell viability assay

Cells were seeded on a six-well plate and transfected with negative control siRNA or H2A.Z.1 siRNA. After transfection, cells were incubated for 72 hr, harvested by trypsinization and stained using trypan blue solution (Sigma, ST. Louis, MO). After staining, cells were counted using hemocytometer (Marienfeld Superior, Lauda-Königshofen, AW).

### Cell proliferation assay

3-(4,5-dimethylthiazol-2-yl)-5-(3-carboxymethoxyphenyl)-2-(4-sulfophenyl)-2H-tetrazolium (MTS) assays were performed to determine relative cell proliferation. Cells were plated in a 12-well plate and transfected with negative control siRNA or H2A.Z.1 siRNA. After transfection, to measure cell proliferation, the cells were incubated in 1/20 diluted 500 ul the cellTiter 96^®^ One Solution Cell Proliferation assay solution (Promega, Madison, WI) every 24 hr. One hour later, cell absorbance was detected by a VICTOR3^™^ Multilabel plate reader (PerkinElmer Inc, Boston, MA) at a wavelength of 490 nm.

### Bromodeoxyuridine (BrdU) incorporation assay

Cells were seeded in a 24-well plate to 40%–50% confluency. Negative control or H2A.Z.1 siRNA was transfected to the seeded cells, and the assay was performed with a BrdU cell proliferation assay kit (Millipore, Billerica, MA) in accordance with the manufacturer's protocol every 24 hr.

### Clonogenic assay

Cells were transfected with H2A.Z.1 siRNA in 60 mm^2^ cell culture plates. After transfection for 24 hr, 2 × 10^3^, 4 × 10^3^, and 6 × 10^3^ cells were reseeded in 6-well plates and incubated for 2 weeks. Next, cells were washed with PBS and fixed with 1% paraformaldehyde for 30 min at room temperature. Fixed cells were stained with 0.5% crystal violet for 1 hr at room temperature. Colonies were counted using a colono-counter program as previously described [21].

### Cell cycle analysis

To determine cell distribution, cells seeded in 60 mm^2^ plates were transfected with negative control and H2A.Z.1 siRNA. After 72 hr incubation, cells were collected by trypsinization. Then, harvested cells were fixed using 70% ethanol, washed in PBS, and resuspended by 200 ul PBS including 1 mg/mL RNase, 0.05% Triton X-100 and 50 ug/mL propidium iodide (BD Biosciences, San Jose, CA, USA). Then, suspended cells were incubated in a 37°C incubator without CO_2_ for 1 hr, and analysis was carried out using the FACSCalibur flow cytometer (BD Biosciences, San Jose, CA, USA) with FLOWJO software (Tree Star, Ashland, OR).

### Apoptosis assay

The Annexin V-FITC Apoptosis Detection Kit I (BD Biosciences, San Diego, CA, USA) was used to measure the level of apoptosis. After transfection for 72 hr, cells were trypsinized, rinsed in PBS, and resuspended using 1 × binding buffer (1 × 10^6^ cells/mL), and 100 ul containing 1 × 10^5^ cells were transferred to 5 ml culture tube. Then, 5 ul of Annexin V-FITC and 5 ul of propidium iodide (PI) solution were added. Cells were incubated at room temperature for 15 min in dark. After incubation, a 400 ul 1 × binding buffer was put to each tube, and apoptotic fractions were detected using the FACSCalibur flow cytometer (BD Biosciences, San Jose, CA, USA) and analyzed using FLOWJO software (Tree Star, Ashland, OR).

### RNA isolation and quantitative real-time polymerase chain reaction (qRT-PCR)

Total RNA was isolated from frozen tissues and cell lines using TRIzol reagent (Invitrogen, Carlsbad, CA, USA) according to the manufacturer's instructions. To synthesize cDNA, a tetro cDNA synthesis kit (Bioline USA Inc., Tounton, MA, USA) was used. For qRT-PCR analysis, reactions were conducted with SensiFAST™ SYBR^®^ No-ROX Kit (Bioline USA Inc., Tounton, MA, USA). The level of GAPDH was used as a loading control. The PCR was monitored in real time using IQ-5 (Bio-Rad) that allowed for checking the threshold cycle (Ct): the exponential amplification time of PCR products. Results show the mean values from triplicate experiments. Relative expression values were normalized to control −2^−(Target Ct−Control Ct)^. The primer sequences for *H2AFZ* amplification were 5′-GCA GTT TGA ATC GCG GTG-3′ (forward) and 5′-GAG TCC TTT CCA GCC TTA CC-3′ (reverse), and GAPDH primer sequences were 5′-ACC AGG TGG TCT CCT CTG AC-3′ (forward) and 5′-TGC TGT AGC CAA ATT CGT TG-3′ (reverse).

### Motility and invasion assay

For *in vitro* cell motility and invasion assay, cell migration was measured using a modified Boyden chamber assay (BD Bioscience, San Jose, CA, USA). For invasion assay, Matrigel (BD Biosciences) was diluted to 0.3 mg/ml concentration with coating buffer (0.01 M Tris, 0.7% NaCl, pH 8.0). Then 100 ul matrigel was coated on upper parts of cell culture insert. After 2 hr incubation at 37°C, the insert was ready to seed. After the insert preparation, cells were plated on the top surfaces of transwell inserts (8-um pore size), and then the inserts were placed in a 24-well plate. Lower wells contained 5% FBS as a chemoattractant. The plate was incubated overnight and stained by Diff-Quik staining kit (Sysmex, Japan). The cells images were captured using an Axiovert 200 inverted microscope (Zeiss, Germany) at ×200 magnification, and the number of cells was counted in three random image fields.

### Wound healing assay

Cells were transfected and incubated for 24 hr in 60 mm^2^ cell culture plates. Then, cells were trypsinized, 1 × 10^6^ cells per well were seeded in a 6-well cell culture plate. After overnight incubation, cell monolayers were scraped with a sterile micropipette tip. Initial gap widths 0 hr after scratching and residual gap widths 24 hr after scratching were photographed using a photomicrograph.

### Gene set enrichment analysis

To investigate the underlying mechanisms of H2A.Z.1, gene set enrichment analysis (GSEA) was performed using an *H2AFZ*-correlated gene set that was identified in a large HCC cohort study (GSE16757). Given a data set in which genes were rank-ordered by the correlation of their expression levels by phenotype of interest, the basic GSEA test provides a score that quantifies the degree of enrichment of a given gene set at the top (positive correlated) or bottom (negative correlated) of the rank-ordered data set. The proximities of gene sets were measured using Kolmogorov-Smirnoff (KS) scores (a higher score corresponded to greater proximity). Observed KS scores were compared with the distribution of 1000 permuted KS scores for all gene sets to assess significance.

### Molecular concept map

ConceptGen, an open-source gene set enrichment testing and concept mapping tool, was used to create a network graph to visualize the interconnectivity among sets of genes (concepts) (http://conceptgen.ncibi.org/core/conceptGen/index.jsp). To provide the functional relevance of an *H2AFZ*-specific gene set in HCC, we first performed with the *H2AFZ*-correlated gene set identified in a large HCC cohort study (GSE16757) by assessing their molecular correlations using the ConceptGen Molecular Concept Map (MCM), which contains over 20,000 molecular concepts that comprised 14 biological knowledge types, for enrichment by disproportionate overlap using a modified Fisher's extract test [16]. MCM analysis of 477 H2AFZ-correlated gene (*P* < 0.01, *r* > 0.4 or *r* < −0.4) with other cancer-related gene sets was performed, and an enrichment network was generated.

### Tumor xenograft assay

For *in vivo* tumor xenograft assay, 1 × 10^7^ cells of the transfected Hep3B (N.C or siH2A.Z.1 siRNA, respectively) were mixed with 0.2 ml complete media and the cell suspension was subcutaneously injected into the flank of 4-week-old male nude mice. After injection, mice were examined two or three times per week for tumor formation at the sites of injection. After 50 days of inoculation, the mice were sacrificed for obtaining tumor tissue. For xenograft imaging, the mice were injected with 100 ul Fluorescent imaging agent (MMPSensTM 750 FAST) (PerkinElmer Inc, Boston, MA, USA) before imaging. After overnight, the mice that fluorescent imaging agent was injected were placed in the light-tight imaging chamber of IVIS Lumina XRMS (PerkinElmer Inc, Boston, MA, USA). A gray-scale reference image was obtained followed by the acquisition of a bioluminescent image. The acquisition time ranged from 1 to 2 minutes.

### Statistical analysis

All experiments were performed at least three times, and all samples were analyzed in triplicate. Results are presented as mean ± standard deviation (SD). Statistical differences between groups were evaluated by unpaired two-tailed Student's *t*-test using Graphpad™ 5.0 (GraphPad software Inc., San Diego, CA, USA). *P*-values less than 0.05 were considered statistically significant.

## SUPPLEMENTARY MATERIALS FIGURES AND TABLES


