# Supplementary material for: Oncogenic potential of histone-variant H2A.Z.1 and its regulatory role in cell cycle and epithelial-mesenchymal transition in liver cancer
Source: Oncotarget. 2016 Feb 4;7(10):11412–23. doi: 10.18632/oncotarget.7194 (PMC4905482; doi:10.18632/oncotarget.7194)
Supplement: Supplementary file 1 [file oncotarget-07-11412-s001.pdf]

Oncogenic potential of histone-variant H2A.Z.1 and its regulatory role in cell cycle and epithelial-mesenchymal transition in liver cancer

Supplementary Materials

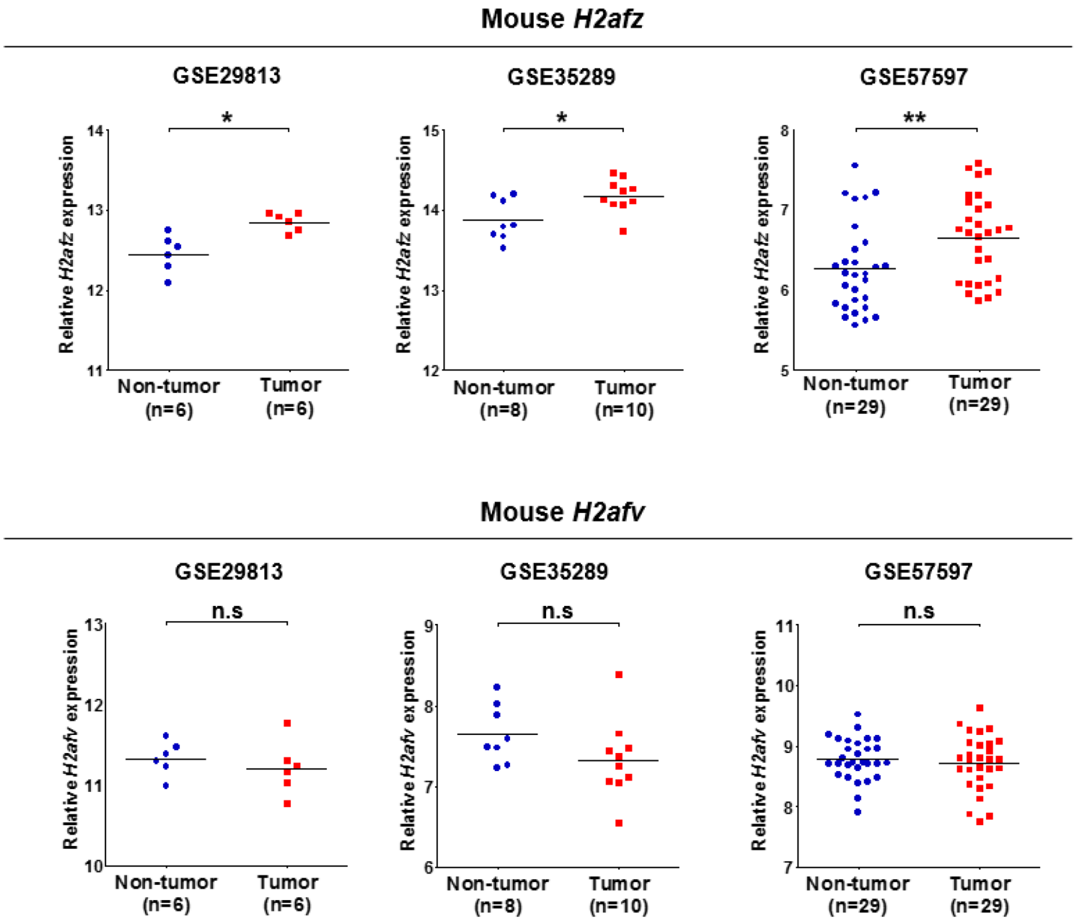

Supplementary Figure S1: Aberrant expression of *H2afz* in a subset of mouse HCC. Analysis of microarray data from the Gene Expression Omnibus (GEO) database. GSE data sets of GSE29813, GSE35289, and GSE57597 showed that *H2afz* was significantly overexpressed in mouse HCC. Upper panel: *H2afz* mRNA expression in mouse HCC. Lower panel: *H2afv* mRNA expression in mouse HCC (mean ± S.D., **$P < 0.01$, *$P < 0.05$, versus Non-tumor).

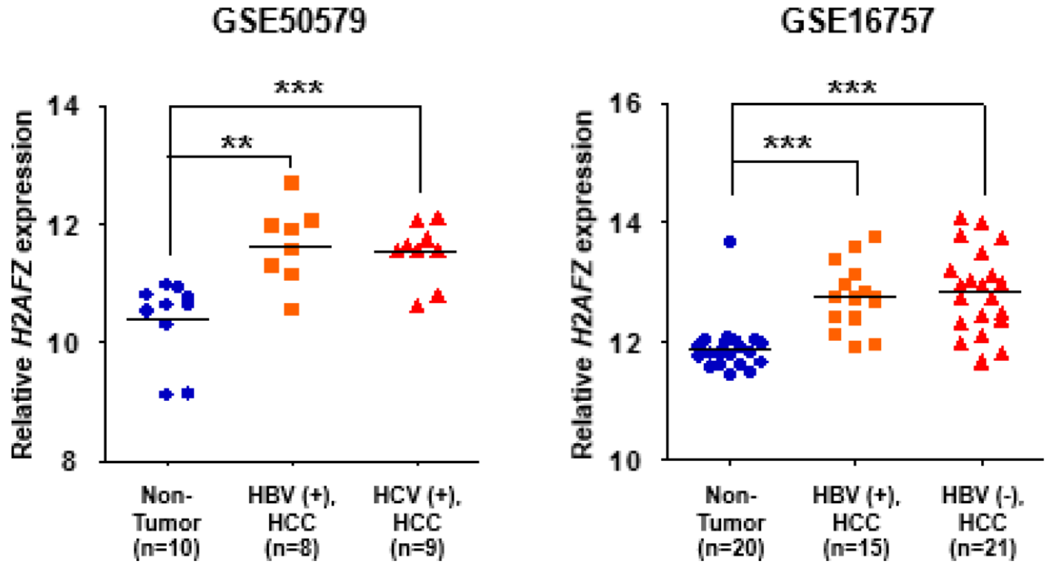

Supplementary Figure S2: The *H2AFZ* gene expression in HCC patients with different etiologies. GSE50579 and GSE16757 showed that *H2AFZ* was significantly overexpressed regardless HBV or HCV etiology. Comparison of *H2AFZ* expression in HCC patients with HBV(+) and HCV(+) versus non-tumor (GSE50579) or HBV(+) and HBV(−) versus non-tumor (GSE16757) (mean ± S.D., ***$P < 0.001$, **$P < 0.01$, versus Non-tumor).

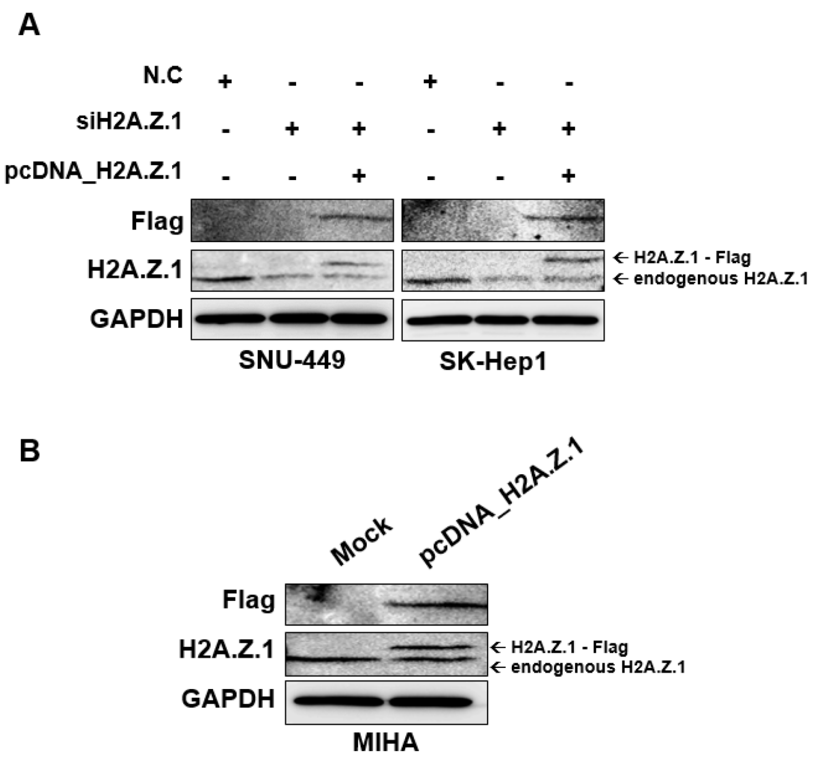

Supplementary Figure S3: Western blot analysis of H2A.Z.1 expressing plasmid in liver cell lines. (**A**) After 48 hr transfection, cell lysate was obtained and western blot analysis was performed to specific antibodies. (**B**) Each pcDNA3.1_Mock or pcDNA3.1_H2A.Z.1 plasmid was transfected into MIHA cells. After 48 hr incubation, the western blot analysis was performed for detection of endogenous or exogenous H2A.Z.1 protein.

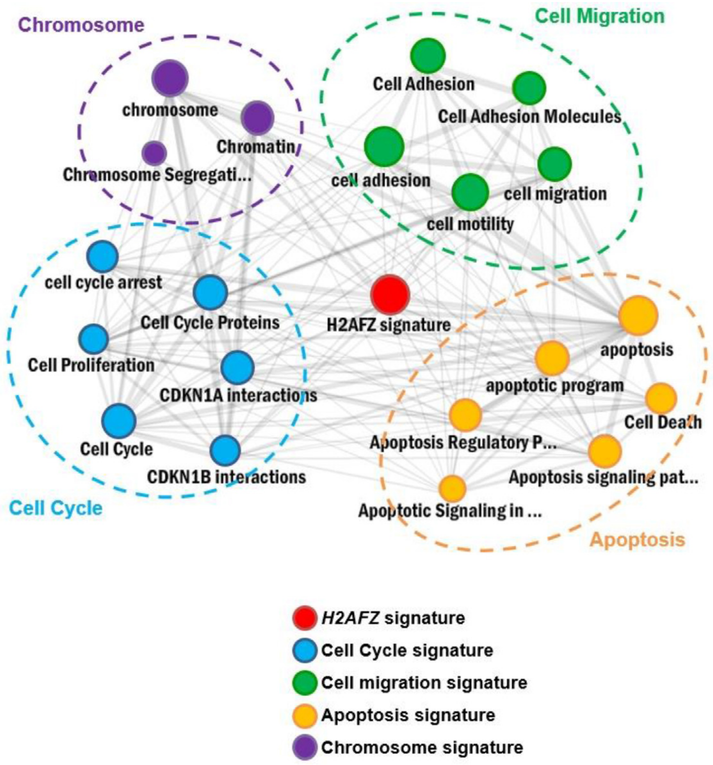

Supplementary Figure S4: An enrichment network analysis that linked *H2AFZ* signatures with cellular pathways. *H2AFZ*-associated genes were analyzed by using the molecular concept map (ConceptGen). Four major molecular concepts were indicated by colored circles.

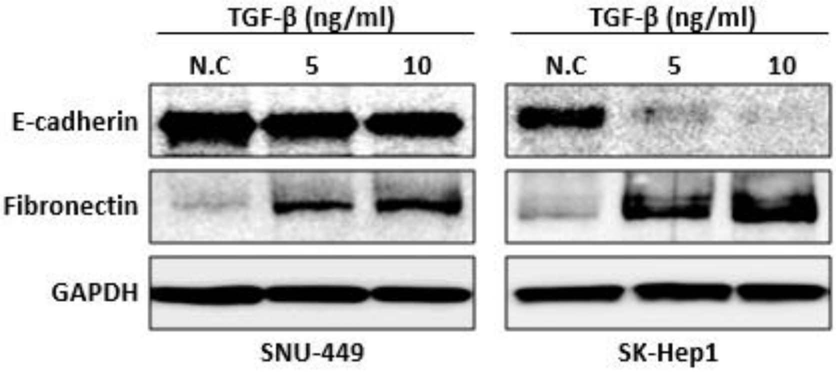

Supplementary Figure S5: Effect of TGF-β on E-cadherin and fibronectin expression in liver cancer cells. The western blot analysis was performed and the protein levels of E-cadherin and fibronectin were detected by specific antibodies. The GAPDH was used for a loading control.

Supplementary Table S1: *H2AFZ* and *H2AFV* expression in HCC GEO datasets

GEO No.	GSE14520		GSE16757		GSE22058		GSE36376	
cohort	(N = 220, T = 225)		(N = 20, T = 100)		(N = 97, T = 100)		(N = 193, T = 240)	
	Fold change	*P* value	Fold change	*P* value	Fold change	*P* value	Fold change	*P* value
H2AFZ	2.771	$p < 0.001$	2.126	$p < 0.001$	1.535	$p < 0.001$	2.279	$p < 0.001$
H2AFV	1.154	$p < 0.001$	1.015	ns	1.112	$p < 0.01$	1.098	$p < 0.001$

Supplementary Table S2: *H2AFZ*-associated functional annotation cluster list

Cluster No.	Title	Term	Count	*p* value
1	**Cell Cycle**	cell cycle phase	45	$p < 0.001$
		cell cycle	58	$p < 0.001$
		M phase	36	$p < 0.001$
		cell cycle process	47	$p < 0.001$
		mitotic cell cycle	36	$p < 0.001$
		organelle fission	25	$p < 0.001$
		mitosis	24	$p < 0.001$
		nuclear division	24	$p < 0.001$
		M phase of mitotic cell cycle	24	$p < 0.001$
		cell division	23	$p < 0.001$
2	**DNA Repair**	DNA metabolic process	45	$p < 0.001$
		DNA repair	26	$p < 0.001$
		response to DNA damage stimulus	30	$p < 0.001$
		cellular response to stress	33	$p < 0.001$
3	**Nuclear Lumen**	organelle lumen	82	$p < 0.001$
		nucleoplasm	51	$p < 0.001$
		intracellular organelle lumen	80	$p < 0.001$
		membrane-enclosed lumen	82	$p < 0.001$
		nuclear lumen	66	$p < 0.001$

**4**	**Chromosome**	chromosome	44	$p < 0.001$
		chromosomal part	37	$p < 0.001$
		condensed chromosome	20	$p < 0.001$
		chromosome, centromeric region	18	$p < 0.001$
		kinetochore	13	$p < 0.001$
		condensed chromosome, centromeric region	12	$p < 0.001$
		condensed chromosome kinetochore	11	$p < 0.001$
		chromosome segregation	11	$p < 0.001$
		non-membrane-bounded organelle	88	$p < 0.001$
		intracellular non-membrane-bounded organelle	88	$p < 0.001$
**5**	**Mitosis**	mitotic cell cycle	36	$p < 0.001$
		interphase of mitotic cell cycle	15	$p < 0.001$
		interphase	15	$p < 0.001$
		G1/S transition of mitotic cell cycle	8	$p < 0.001$
**6**	**Macromolecule Assembly**	macromolecular complex subunit organization	39	$p < 0.001$
		macromolecular complex assembly	35	$p < 0.001$
		cellular macromolecular complex subunit organization	23	$p < 0.001$
		cellular macromolecular complex assembly	19	$p < 0.001$
		protein complex biogenesis	25	$p < 0.01$
		protein complex assembly	25	$p < 0.01$
**7**	**Meiosis**	DNA recombination	13	$p < 0.001$
		meiotic cell cycle	12	$p < 0.001$
		M phase of meiotic cell cycle	11	$p < 0.001$
		meiosis	11	$p < 0.001$
		reciprocal meiotic recombination	4	$p < 0.05$
		meiosis I	4	n.s
**8**	**Microtubule Process**	microtubule cytoskeleton organization	14	$p < 0.001$
		spindle organization	8	$p < 0.001$
		microtubule-based process	17	$p < 0.001$
		cytoskeleton organization	19	$p < 0.05$

**9**	**ATPase Activity**	DNA-dependent ATPase activity	9	$p < 0.001$
		ATPase activity	20	$p < 0.001$
		ATPase activity, coupled	15	$p < 0.01$
**10**	**DNA Replication**	replication fork	8	$p < 0.001$
		DNA replication factor C complex	4	$p < 0.001$
		nucleotide-excision repair, DNA gap filling	5	$p < 0.001$
		DNA clamp loader activity	3	$p < 0.001$
		protein-DNA loading ATPase activity	3	$p < 0.01$
		nucleotide-excision repair	6	$p < 0.01$

Supplementary Table S3: Positive enrichment gene sets of *H2AFZ* high

No.	Gene Sets	SIZE	ES	NES
1	REACTOME_CELL_CYCLE	42	0.65	1.35
2	REACTOME_CELL_CYCLE_MITOTIC	38	0.64	1.32
3	REACTOME_MITOTIC_M_M_G1_PHASES	23	0.66	1.28
4	REACTOME_MITOTIC_PROMETAPHASE	16	0.62	1.24
5	REACTOME_DNA_REPLICATION	28	0.6	1.2
6	REACTOME_ADAPTIVE_IMMUNE_SYSTEM	17	0.4	1.16
7	REACTOME_IMMUNE_SYSTEM	25	0.24	0.76
8	REACTOME_HEMOSTASIS	22	0.18	0.55
9	REACTOME_METABOLISM_OF_LIPIDS_AND_LIPOPROTEINS	16	0.19	0.53
